# Propensity score matched comparison of lymph node upstaging in early-stage lung cancer: open versus minimally invasive surgery with standardized lymphadenectomy

**DOI:** 10.1186/s13019-025-03346-5

**Published:** 2025-01-27

**Authors:** Julia Zimmermann, Julia Walter, Valentina Pfeiffer, Julia Kovács, Gökçe Yavuz, Johannes Schön, Mircea Gabriel Stoleriu, Christian Ketscher, Niels Reinmuth, Rudolf A. Hatz, Amanda Tufman, Christian P. Schneider

**Affiliations:** 1 Division of Thoracic Surgery, LMU University Hospital, LMU Munich and Asklepios Lung Clinic, Gauting, Germany; 2 Department of Internal Medicine V, LMU University Hospital, LMU, Munich, Germany; 3 Department of Thoracic Oncology, Asklepios Lung Clinic, Gauting, Germany; 4 Comprehensive Pneumology Center Munich, German Center of Lung Research (DZL), 81377 Munich, Germany; 5 Division of Thoracic Surgery, Ludwig-Maximilians-University, Marchionini Street 15, 81377 Munich, Germany

**Keywords:** Non-small cell lung cancer, Lymphadenectomy, Propensity score matching, Tumor staging, Thoracotomy, Video-assisted thoracoscopic surgery, Survival

## Abstract

**Background:**

Lymph node upstaging represents a quality criterion for standardized lymphadenectomy in lung cancer surgery. The aim of the study was to compare whether the quality of standardized lymphadenectomy in lung cancer surgery is comparable in minimally invasive (video-assisted thoracoscopic surgery) and the open approach (thoracotomy). Furthermore, factors associated with lymph node upstaging were assessed, as was its impact on overall survival and progression-free survival.

**Methods:**

This retrospective study reviewed data of all patients undergoing lobectomy at the Lung Tumor Center Munich between 2011 and 2020. Inclusion factors were non-small cell lung cancer without nodal involvement (N0) or metastasis (M0) and standardized lymphadenectomy. A propensity score matched analyses was performed. Frequency of categorical outcomes was compared with Chi [2]-test, mean values with t-test. We used logistic and Cox regression models to assess factors associated with upstaging, overall survival and progression-free survival, restrictively.

**Results:**

Of 1691 patients undergoing lobectomy, 637 met our inclusion criteria. After propensity score matching 198 patients remained in each group. Univariate analysis showed no significant difference in lymph node upstaging between the two groups. (*p* = 0.12). Overall affected lymph nodes (*p* = 0.45) and overall affected lymph node stations (*p* = 0.26) were not significantly different. Multivariate Cox regression analysis showed that overall survival and progression free survival were also independent of the surgical approach. L1 status was the only factor associated with progression-free survival.

**Conclusion:**

Minimally invasive approaches achieves comparable lymph node upstaging in patients undergone standardized lymphadenectomy.

## Introduction

Complete lymphadenectomy remains one of the most important components in curative intent resection of lung cancer. Accurate tumor staging through complete lymphadenectomy is primarily used for further therapy planning and results in potential survival benefit [1, 2]. 

An important quality criterion of lymphadenectomy is postoperative lymph node (LN) upstaging, which is defined as the unexpected pathological finding of metastasis in hilar (N1) or mediastinal (N2) LNs [3]. 

The International Association for the Study of Lung Cancer (IASLC) sponsored efforts to standardize lymphadenectomy procedures, resulting in a report on the international workshop on thoracic staging in 1996, which was taken up by the IASLC in 2005 [4, 5]. After 15 years, these standardized procedures were revised by the Commission on Cancer [6]. In the latest edition of the Optimal Resources for Cancer Care (2020 standards), which was first released in February 2023, it is specified that the surgical pathology report associated with any curative intent pulmonary resection for primary lung malignancy must report the oncologic status of LNs for at least one hilar station (pN1) and at least three distinct mediastinal stations (pN2) [6]. A National Cancer Database query and a multi-institutional prospective study showed that adherence to this nodal sampling standards resulted in a significantly better overall survival, improved lymph node yield and stratification of postoperative stage-specific survival curves [7, 8], but adherence to this recommendation is variable.

Unfortunately, in many studies lymphadenectomy has not been performed according to the guidelines. A standardized procedure for lymphadenectomy is the only way to compare minimally invasive surgery (video-assisted thoracoscopic surgery - VATS) and thoracotomy (open surgery) with regard to LN upstaging.

This study aims to compare standardized lymphadenectomy in thoracotomy and VATS to evaluate differences in LN upstaging and survival outcomes.

## Methods

### Study design, patient cohort and data collection

In this retrospective analysis, we used data of all lung cancer patients with preoperative N0-status undergoing lobectomy through thoracotomy or VATS at the Lung Tumor Center Munich between 2011 and 2020. Only patients with standardized lymphadenectomy were included, as defined by the Commission on Cancer [6].

Preoperatively, all patients were staged according to the current National comprehensive Cancer Network (NCCN) guidelines and were discussed at the specific tumor board. Patients underwent pre- or intraoperative bronchoscopy, pathological lymph node evaluation and FDG-PET/CT scan. Meanwhile, the method of choice for lymph node evaluation was EBUS-TBNA. In the past, a mediastinoscopy was performed in cases of suspected lymph node involvement. This is still the method of choice for an EBUS-TBNA negative for malignancy in a clinically (FDG-PET/CT and/or CT) positive mediastinum. Patients with clinical stage II and expected N0 status also receive a cranial MRI [9].

Our exclusion criteria were patients with wedge resection, segmentectomy or pneumonectomy, conversion to thoracotomy, patients with preoperative nodal involvement at diagnosis (N1, N2 and N3), patients with distant metastases at diagnosis, and patients with missing pathological N status.

**Fig. 1:**
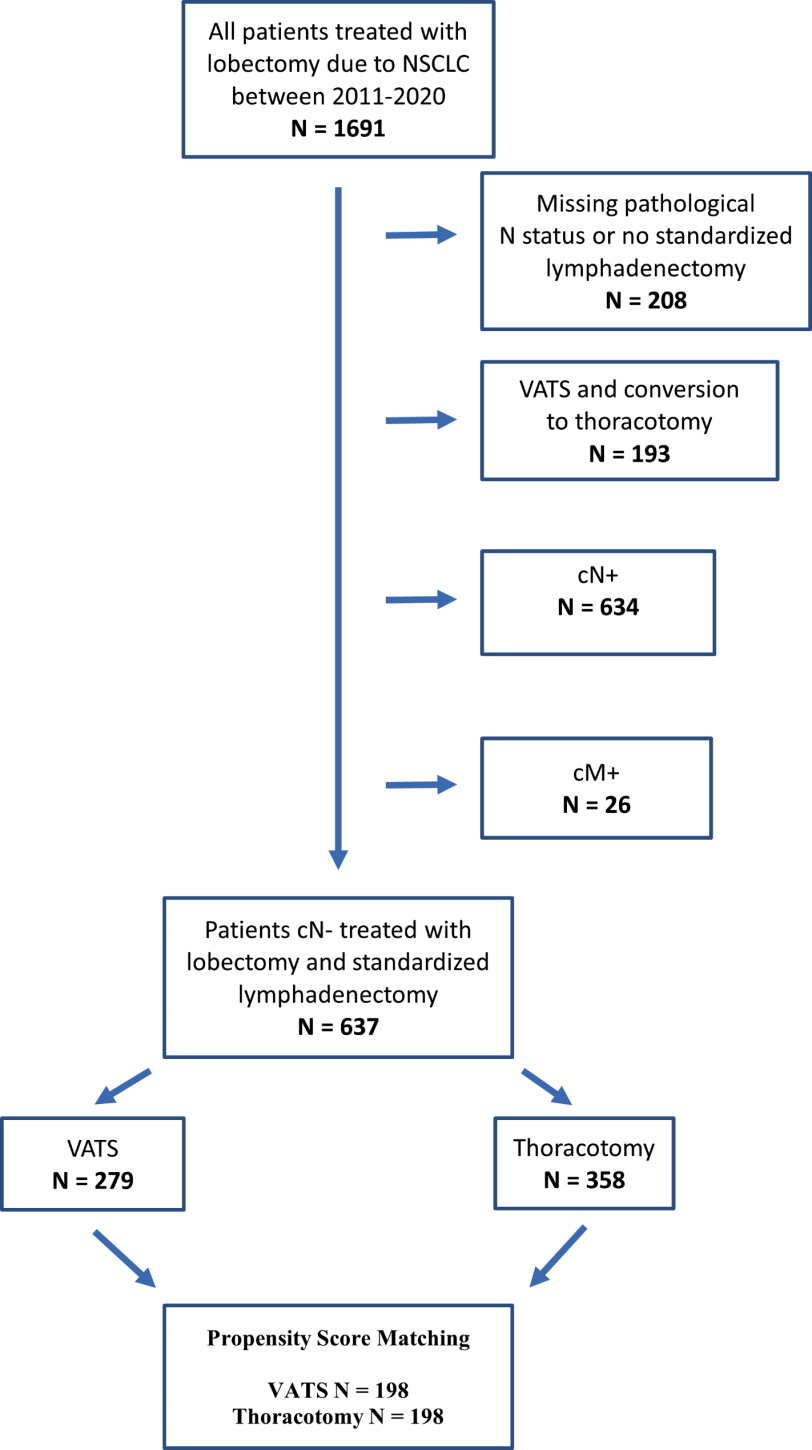
Patient population. cM + = clinical positive distant metastases, cN + = clinical positive lymph nodes, N Status = lymph node status, NSCLC = non-small cell lung cancer, VATS = video-assisted thoracic surgery

A detailed representation of the inclusion and exclusion criteria of the patient population is shown in Fig. 1.

All information in the dataset was extracted from electronic patient records and archived charts. This data included information about patient characteristics such as age at resection, sex, performance status according to the American Society of Anesthesiologists risk classification (ASA), comorbidities, body mass index (BMI), forced expiratory volume in 1 s (FEV1%), year of lobectomy and smoking status. Tumor characteristics covered clinical and pathological tumor stage, histological type, tumor location, as well as tumor grading, lymphovascular space invasion (L-status) and vascular invasion (V-status). Additionally, we documented the number of assessed and the number of affected LNs for each individual LN station.

### Definition of standardized lymphadenectomy and upstaging

Standardized lymphadenectomy was defined as assessing at least one LN station in N1 and assessing at least three LN stations in N2 during surgery, as defined by the Commission on Cancer [6]. 

As only patients with preoperative N0-status were included, LN upstaging was defined as either having an affected N1- or N2-LN after surgery through lymphadenectomy. We distinguished between upstaging from N0 to N1 and upstaging from N0 to N2.

In addition to LN upstaging we also compared the frequency of assessment of LN stations as well as the number of assessed LNs in each station and overall. We grouped adjacent mediastinal LN stations to paratracheal (#2, #4), and to pulmonary ligament/paraesophageal (#8, #9).

### Categorization of variables and handling of missing data

We categorized histological types into adenocarcinoma (ACC), squamous-cell carcinoma (SCC), and neuroendocrine carcinoma (including carcinoids and large-cell neuroendocrine carcinomas) (NEC). All other histological types were summarized under the category “other histology”.

As BMI was missing in 7 patients we used multiple imputation to fill in the missing values, with the methods described in the next paragraph.

### Statistical analysis

We applied propensity score matching (PSM) to compare upstaging, overall survival (OS), and progression-free survival (PFS) between patients undergoing VATS and thoracotomy. The primary goal of using PSM was to mitigate potential selection bias by balancing baseline characteristics between the two surgical groups, as patients undergoing VATS may differ systematically from those undergoing thoracotomy in ways that could confound the results. For the propensity score matching, we utilized the nearest neighbor matching method with a caliper of 0.1 to ensure that matched pairs were as similar as possible, minimizing potential biases while retaining a sufficient number of matched patients for analysis. The variables selected for the propensity score model were chosen based on a combination of expert opinion, a comprehensive review of the existing literature, and an assessment of the standardized mean difference (SMD). Expert opinion was sought to identify key clinical factors that could influence the choice of surgical approach and that are likely to impact upstaging, OS, and PFS. A literature review was conducted to further inform this process, ensuring that the selected variables reflected those typically associated with these outcomes in similar patient populations. These factors included age, sex, comorbidities, tumor size, stage of disease, and performance status, among others. To assess the balance between the groups before and after matching, we used the standardized mean difference (SMD), which quantifies the difference in means between the groups, standardized by the pooled standard deviation. An SMD greater than 0.1 indicates a notable imbalance between groups, while an SMD below 0.1 suggests adequate balance. Patient characteristics are presented as mean values with standard deviation (SD) for metric variables and absolute and relative frequencies for categorical variables. They were compared between thoracotomy and VATS patients using Students t-test for metric variables, and Chi [2]-test or fisher-exact test, when cell numbers were < 6, for categorical variables. Statistical significance for these comparisons was determined using two-sided *p*-values with alpha errors < 0.05. Multiple imputation of BMI was performed using the R package “mice,” which applies a conditional multiple imputation method. The variables used in the imputation process—age, sex, BMI, and all assessed comorbidities—were selected because they are known to be closely related to both BMI and the outcomes of interest in this study. Age and sex are fundamental demographic factors that often influence body mass index and can confound relationships between BMI and clinical outcomes. Comorbidities, such as hypertension, diabetes, and cardiovascular disease, are also frequently associated with BMI and can impact both the likelihood of certain surgical approaches and post-operative outcomes. By including these variables in the imputation process, we aimed to improve the accuracy of the imputed BMI values and reduce bias in the analysis, ensuring that missing data did not unduly affect the validity of our results.We used a mixed effects logistic regression model including the match-ID as a random effect to assess the association between upstaging and type of surgery. In this model the number of assessed LNs, tumor grading, L- and V-status were included as possible additional confounding variables not chosen in the PSM. The number of assessed LNs was considered a post-treatment variable, while tumor grading and L- and V-status did not show significant imbalance prior to matching. They were chosen based on their clinical relevance and their potential impact on the likelihood of upstaging.The significance of these variables was assessed using the Wald test. To assess the association of OS, PFS and surgical approach we used Cox regression models including the match-ID as a cluster variable to account for the matched nature of the data. The models were adjusted for additional confounders including CCI score, tumor grading, and L- and V-status as confounders. By adjusting for these additional variables, we aimed to control for potential confounding factors that could bias the relationship between surgical approach and survival outcomes, thereby providing a more accurate estimate of the effect of surgery on OS and PFS.

Data analysis was performed using R Version 4.0.0 and RStudio Version 1.4. Tables and the figure were created in RStudio and Microsoft Excel.

## Results

### Patient population

In total, 1691 patients underwent lobectomy at our center between 2011 and 2020. After selection according to our inclusion and exclusion criteria, the data of 279 patients with VATS and 358 patients with thoracotomy were analyzed. The following variables were selected to be used in the PSM as they are associated with the relevant outcomes and surgical approach and showed a SMD of greater than 0.1: age, FEV1% predicted at baseline, BMI, tumor size, sex, ASA, histological type, location of the tumor, diabetes with end-organ-failure, coronary heart disease, and year of resection. PSM resulted in a matched cohort of 198 patients with VATS and 198 patients with thoracotomy. A detailed representation of the composition of the matched patient population is shown in Fig. 1. Characteristics of the final matched study population grouped by VATS and thoracotomy are summarized in Table 1 and shows that the matched sample is well balanced between the two groups.

**Table 1:** Patient and tumor characteristics of study population

	VATS (*n* = 198)		thoracotomy (*n* = 198)		*p*-value	smd
	mean	sd	mean	sd		
**age in years**	66.8	10.0	67.4	10.4	0.57	0.06
**FEV1%**	84.6	18.9	84.2	18.6	0.85	0.02
**BMI**	25.8	4.8	25.6	4.5	0.66	0.04
**tumor size in cm**	2.8	1.5	2.9	1.9	0.40	0.09

	n	%	n	%	*p*-value	smd
**sex**						
male	87	43.9%	89	44.9%		
female	111	56.1%	109	55.1%	0.9194	0.02
**current smoker**						
yes	55	27.8%	58	29.3%		
no	141	71.2%	139	70.2%		
unknown	2	1.0%	1	0.5%	0.87	0.07
**ASA**						
1	4	2.0%	4	2.0%		
2	58	29.3%	56	28.3%		
3	126	63.6%	128	64.6%		
4	3	1.5%	3	1.5%		
unknown	7	3.5%	7	3.5%	0.990	0.02
**histological type**						
adenocarcinoma	128	64.6%	128	64.6%		
NEC	21	10.6%	21	10.6%		
SCC	45	22.7%	44	22.2%		
other	4	2.0%	5	2.5%	0.989	0.04
**location**						
middle lobe	14	7.1%	17	8.6%		
upper lobe	116	58.6%	116	58.6%		
lower lobe	68	34.3%	65	32.8%	0.84	0.06
**Tumor grading**						
1	11	5.6%	14	7.1%		
2	105	53.0%	102	51.5%		
3	70	35.4%	62	31.3%		
4	1	0.5%	1	0.5%	0.85	0.08
unknown	11	5.6%	19	9.6%		
**Lymphovascular space invasion (L0**,** L1)**						
0	146	73.7%	145	73.2%		
1	42	21.2%	47	23.7%	0.71	0.05
unknown	10	5.1%	6	3.0%		
**Vascular invasion (V0**,** V1)**						
0	154	77.8%	158	79.8%		
1	34	17.2%	34	17.2%	1.00	0.01
unknown	10	5.1%	6	3.0%		
**Residual tumor classification (R0**,** R1)**						
0	198	100.0%	197	99.5%		
1	0	0.0%	1	0.5%	1	0.10

Patient and tumor characteristics of lung cancer patients with lobectomy and standardized lymphadenectomy stratified by surgical approach. Means with standard deviation of numerical variables and absolute and relative frequency of categorical variables

VATS = video-assisted thoracoscopic surgery, sd = standard deviation, FEV1 = Forced Expiratory Volume in 1 s, BMI = body mass index, ASA = American Society of Anesthesiologists risk classification, NEC = neuroendocrine carcinoma, SCC = squamous-cell carcinoma

### Upstaging of nodal status after surgical resection

**Fig. 2:**
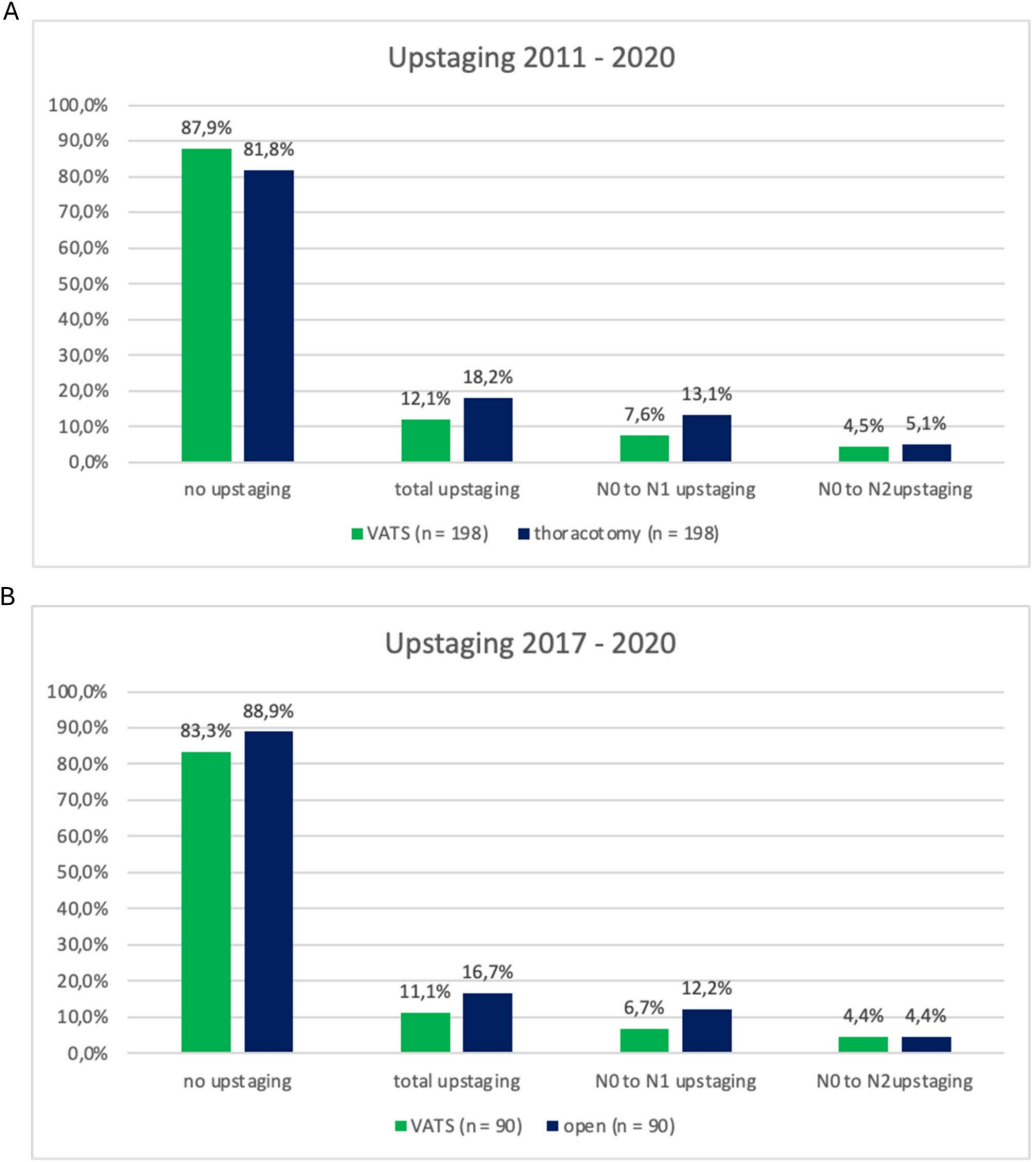
Upstaging stratified by surgical approach. Upstaging of N stage after lobectomy stratified by type of resection. **A** displays upstaging in all patients (2011 to 2020). **B** shows results in patients with lobectomies between 2017 and 2020. *P*-values from Chi^2^-test. VATS = video-assisted thoracoscopic surgery

As shown in Fig. 2A, pathological LN upstaging was not significant regading the surgical approach. We had an overall rate of 12.1% in the VATS group compared to 18.2% in the thoracotomy group (*p* = 0.12). This difference in the overall rate of upstaging between VATS and thoracotomy is mainly explained by upstaging from N0 to N1 (7.6% vs. 13.1%). Upstaging from N0 to N2 was similar in both groups (4.5% vs. 5.1%).

### Nodal assessment during surgery

The frequency of assessment of the hilar nodal station (#10) was significantly higher in thoracotomy (77.3%) compared to VATS (66.2%) (p 0.02). We did not find significant differences in the frequency of nodal assessment regarding all other LN stations. The average overall number of N1 LN assessed was significantly higher in thoracotomy (7.4, sd = 4.6) compared to VATS (5.8, sd = 4.0). This was mainly driven by the significant difference in hilar LNs (*p* = 0.043). Intrapulmonary LNs were significantly higher in thoracotomy, but cannot be influenced by the surgical approach, because they are located in the lung parenchyma of the lung lobe that is removed. In N2 the average overall number of assessed LNs was significantly higher in thoracotomy (10.2, sd = 7.0) compared to VATS (8.4, sd = 4.8) (p 0.003). While we found significantly higher numbers of assessed LNs for paratracheal (*p* = 0.033) and pulmonary ligaments/paraesophageal stations (*p* = 0.03) in thoracotomy than in VATS, no significant difference was shown for the aortopulmonary window (*p* = 0.47) and subcarinal (*p* = 0.15).

No significant difference was determined for the number of overall, N1 and N2 affected LN stations as well as overall, N1 and N2 affected LNs.

A complete overview on the frequency of assessed stations and the number of assessed LNs stratified by surgical approach can be found in Table 2.

**Table 2:** Frequency of assessed lymph nodes and number of assessed lymph nodes by stations and surgical approach

	VATS(= 198)		thoracotomy (*n* = 198)			
	n	%	n	%		*p*-value
**frequency of stations assessed**						
**N1**	*472*	*59.6%*	*502*	*63.4%*		
hilar (#10)	131	66.2%	153	77.3%	**0.02**	
interlobar (#11)	168	84.8%	167	84.3%	1.00	
lobar (#12)	13	6.6%	18	9.1%	0.45	
intrapulmonary	160	80.8%	164	82.8%	0.69	
**N2**	*554*	*69.9%*	*567*	*71.6%*		
paratracheal (#2,#4)	125	63.1%	129	65.2%	0.75	
aortopulmonary window (#5,#6)	76	38.4%	76	38.4%	1.00	
subcarinal (#7)	182	91.9%	186	93.9%	0.56	
pulmonary ligament/paraesophageal (#8,#9)	171	86.4%	176	88.9%	0.54	
	**mean**	**sd**	**mean**	**sd**	***p*** **-value**	
**# of LN assessed**						
**N1**	5.8	4.0	7.4	4.6	**0.001**	
hilar (#10)	1.5	1.8	1.9	1.8	0.043	
interlobar (#11)	2.2	1.9	2.4	2.1	0.23	
lobar (#12)	0.2	0.9	0.2	0.9	0.79	
intrapulmonary	2.6	2.3	3.5	3.8	**0.007**	
**N2**	8.4	4.8	10.2	7.0	**0.003**	
paratracheal (#2,#4)	2.9	3.4	3.8	4.5	**0.033**	
aortopulmonary window (#5,#6)	1.1	1.9	1.2	2.1	0.77	
subcarinal (#7)	2.6	2.2	3.0	2.7	0.15	
pulmonary ligament/paraesophageal (#8,#9)	2.0	1.7	2.4	2.2	0.03	
**overall stations assessed**	6.1	1.1	6.5	1.2	**0.002**	
**overall LN count**	15.5	6.8	19.0	9.2	**< 0.0001**	
	**mean**	**sd**	**mean**	**sd**	***p*** **-value**	
**# of affected stations**						
overall	0.21	0.73	0.30	0.77	0.26	
N1 LN	0.14	0.44	0.22	0.55	**0.09**	
N2 LN	0.08	0.40	0.08	0.35	1.00	
**# of affected LN**						
overall	0.38	1.64	0.51	1.66	0.45	
N1 LN	0.24	1.00	0.40	1.31	0.16	
N2 LN	0.15	0.87	0.11	0.51	0.57	

Frequency of lymph nodes stations assessed during surgery, number of assessed lymph nodes, and number of affected lymph nodes and stations by surgical approach. Absolute and relative frequency of stations and mean number of assessed lymph nodes with standard deviation. *P*-values from Chi [2]-test for frequencies and Students t-test for number of lymph nodes and stations

VATS = video-assisted thoracoscopic surgery, sd = standard deviation

### Multivariate regression analysis

In the multivariate logistic regression analysis with regard to upstaging (Table 3) the surgical approach showed no significant influence (OR = 0.95, *p* = 0.38) as well as all other selected factors.

**Table 3:** Results from logistic regression analysis of likelihood of upstaging

	OR	beta	se	z-value	*p*-value
VATS vs. thoracotomy	0.95	-0.05	0.04	-1.31	0.38
number of assesed lymph nodes	1.04	0.04	0.08	0.50	0.17
grade 2 vs. grade 1	1.01	0.01	0.08	0.16	0.62
grade 3 vs. grade 1	1.01	0.01	0.08	-0.45	0.87
grade unknown vs. grade 1	0.96	-0.05	0.10	1.44	0.65
L1 vs. L0	1.09	0.08	0.06	0.40	0.15
V1 vs. V0	1.03	0.025	0.06	-0.01	0.69

Results from multivariate logistic regression analysis of upstaging and type of surgery, adjusted by number of assessed lymph nodes, tumor grading and L- and V-status

OR = Odds Ratio, VATS = video-assisted thoracic surgery, L = Lymphovascular space invasion, V = Vascular invasion

In the multivariate cox regression analysis with regard to overall survival, no significant factors were identified either. (Table 4)

**Table 4:** Results from Cox regression analysis of overall survival

	HR	beta	se	z-value	*p*-value
VATS vs. thoracotomy	0.67	-0.40	0.35	-1.12	0.27
CCI score	1.06	0.06	0.07	0.89	0.38
grade 2 vs. grade 1	3.01	1.10	1.03	1.10	0.27
grade 3 vs. grade 1	2.10	0.74	1.07	0.71	0.48
grade unknown vs. grade 1	1.29	0.25	1.42	0.19	0.85
L1 vs. L0	1.52	0.42	0.62	0.70	0.48
V1 vs. V0	1.37	0.32	0.64	0.51	0.61

Results from multivariate Cox regression analysis of overall survival and type of surgery, adjusted by Charlson comorbidity score (CCI score), tumor grading and L, and V status

HR = Hazard Ratio, VATS = video-assisted thoracic surgery, CCI = Charlson comorbidity index, L = Lymphovascular space invasion, V = Vascular invasion

Regarding PFS (Table 5) only L1 vs. L0 status (HR = 4.24, *p* = 0.003) were associated with a significant detrimental effect on PFS.

**Table 5:** Results from Cox regression analysis of overall progression free survival

	HR	beta	se	z-value	*p*-value
VATS vs. thoracotomy	1.01	0.01	0.28	0.03	0.98
CCI score	0.97	-0.03	0.07	-0.40	0.69
grade 2 vs. grade 1	2.76	1.02	1.03	0.97	0.33
grade 3 vs. grade 1	4.13	1.42	1.03	1.37	0.17
grade unknown vs. grade 1	2.44	0.89	1.16	0.76	0.45
L1 vs. L0	4.24	1.44	0.41	2.99	**0.003**
V1 vs. V0	0.62	-0.63	0.45	-1.29	0.20

Results from multivariate Cox regression analysis of overall progression free survival and surgical approach, adjusted by Charlson comorbidity score (CCI score), tumor grading and L, and V status

HR = Hazard Ratio, VATS = video-assisted thoracic surgery, CCI = Charlson comorbidity index, L = Lymphovascular space invasion, V = Vascular invasion

### Upstaging of nodal status after surgical resection in 2017–2020

We had a switch in frequency of lobectomies by surgical approach in 2017 from mainly open to minimally invasive surgery. From this point on, VATS lobectomy was well established at our center and we can assume a high level of expertise. To minimize possible bias in the overall cohort regarding surgical expertise, we performed a sub-analysis of patients who underwent surgery from 2017 to 2020 (Fig. 2B). Upstaging was found in 16.7% of patients with thoracotomy and 11.1% of VATS patients (*p* = 0.39) which was not significant either. Similar to the whole population, upstaging from N0 to N1 was less frequent in VATS compared to thoracotomy, whereas from N0 to N2 it was the same (*p* = 0.44).

## Discussion

In this study we analyzed the LN upstaging for early stage NSCLC after intraoperative standardized lymphadenectomy in a large cohort. Lymphadenectomy is of great importance in lung surgery. The aim of lymphadenectomy is to remove affected lymph nodes that were not thought to be affected preoperatively. Possible LN involvement changes the initial tumour stage and patients receive adjuvant therapy postoperatively, which also has a positive effect on patient survival. Accoording to the guidelines lymphadenectomy should be done standardized.Unfortunately, an adequate standardized lymphadenectomy is often not performed. Some studies published analysed LN upstaging by comparing the different surgical approaches (VATS vs. thoracotomy), but these either had too few overall LN stations assessed or indicated just the overall numbers of stations assessed and hence lacked information about the individual LN stations [10–14]. This was criticised in some of the comments. The guidelines, first published in 1996, prefer to exceed a minimum of six LNs in three hilar and intrapulmonary (N1) and three mediastinal (N2) stations or in the further course LNs from at least one hilar station (N1) and three mediastinal stations (N2) [4, 6]. The American College of Surgeons Oncology Groups randomized trial Z0030, published in 2011, addressed this question and showed that formal lymph node dissection in patients with early-stage non-small cell lung cancer does not improve survival if the systematic and thorough preliminary examination of the mediastinal and hilar lymph nodes is negative [15]. Implementation of this approach in the clinical routine was difficult and the 8th edition of the IASLC Lung Cancer Staging Project demonstrated that more than 50% of resected cases in the staging database were reassigned from R0 to R-undetermined because of incomplete mediastinal lymph node evaluation with significant impact on patient survival [16]. In addition, further analysis of the former Z0030 randomised trial showed that the number of pathological examined lymph nodes was associated with the likelihood of detecting nodal metastasis and survival in the formal lymph node dissection arm [17]. 

The American College of Sugeons Commission on Cancer addressed this issue in the 2020 standards for the Optimal Resources for Cancer Care. Standard 5.8 states that (1) at the time of lung resection hilar and mediastinal lymph nodes should be thoroughly staged even in patients undergoing wedge resection, (2) the pathology report must contain lymph nodes from at least one hilar and three mediastinal stations, and (3) the pathologist must specify all nodal stations in a synoptic format [6]. 

Our study group wanted to carry out an analysis that met the criteria for standardized lymphadenectomy and compare the surgical approaches, VATS and thoracotomy. The study population consisted of patients with assessments in at least one LN station in N1 and at least three LN stations in N2 in the pathological report. On average a total number of 6.1 LN stations were assessed after VATS lobectomy and 6.5 after thoracotomy. The average total LN count was 15.5 in the VATS group and 19.0 in the thoracotomy group. (Table 2) Hence, the definition of standardization is fully met and the average lymph node count in our cohort was comparable to the Z0030 randomized trial from the American College of Surgeons Oncology Group [18]. In this respect, our study differs from others mainly due to an even larger number of removed lymph nodes after standardized lymphadenectomy [19–22]. 

The univariate analysis showed that LN upstaging in thoracotomy group was not significantly higher compared to the VATS lobectomy (18.2% versus 12.1%; *p* = 0.12). (Fig. 2A). The multivariate logistic regression (Table 3) analysis for the likelihood of lymph node upstaging demonstrated no significant parameter either.

We achieved comparable or even higher overall upstaging rates than other published studies [12, 21, 23–25]. However, the difference in upstaging for thoracotomy versus VATS in our analyzes was mainly driven by upstaging from N0 to N1 (13.1 vs. 7.6%), whereas upstaging from N0 to N2 was similar in both groups (5.1% thoracotomy vs. 4.5% VATS). (Fig. 2A).

The fact that the difference in upstaging was maily driven by upstaging from N0 to N1 has already been described in the literature [12]. Regarding the assessment of LN stations, only the hilar #10 station (Table 2) showed a significantly higher frequency of assessment in thoracotomy. This may also explain that N1 stations, were affected significantly more often in thoracotomy, even if there was no significant difference overall. The thoracic surgeons are sometimes more careful with the minimally invasive approach at the hilus to avoid affections of the phrenic nerv, which could explain the difference.

The multivariate cox regression (Table 4) revealed no significant difference for overall survival (HR = 0.67, *p* = 0.27). In our opinion, this underlines the results of our LN upstaging. We were able to show that VATS does not have significantly lower LN upstaging after standardized lymphadenectomy. It can be assumed that the postoperative tumor stage could be determined equally well in the groups and that this also corresponds to the actual tumor stage. As a result, patients can be treated postoperatively according to their tumor stage and the surgical approach therefore has no influence on survival.This is an extraordinarily good result for VATS in particular.

As a critical aspect, we saw that it is difficult to minimize a possible bias due to surgical expertise in our analysis. In 2011, VATS was not yet established and a new technique for all surgeons to learn. It has already been described in the literature that lymphadenectomy improves with the surgeon’s increasing experience and learning curve; more thoracic LN and LN stations are removed [26–28]. This subsequently has an effect on upstaging. We therefore carried out another sub-analysis. From 2017, we performed more VATS lobectomies than thoracotomy lobectomies. It can therefore be assumed that the expertise in VATS lobectomy was improved from this point onwards. The results of the sub-analysis also showed no significant differences with regard to LN upstaging (11.1% VATS and 16.7% thoracotomy; *p* = 0.39).

We have two reasons for this. Firstly, we are of the opinion that, contrary to what is written in the literature, there is no learning curve after performing a standardized lymphadenectomy. We assume that a standardized lymphadenectomy already places high demands on the surgeon and is therefore only performed by experienced surgeons. This also explains the high total number of LN and LN stations represented by our data. As a result, after a standardized lymphadenectomy, the postoperative tumor stage also corresponds to the actual tumor stage. On the other hand, we have a slightly lower upstaging in the sub-analysis than in the overall cohort. This can be explained by the improved preoperative staging that has developed over the years. In the case of large tumors and/or conspicuous LNs, all patients receive a preoperative FDG-PET/CT and an EBUS-TBNA. This has made it possible to detect pre-existing LN involvement in more patients preoperatively. Patients with preoperative positive N-status were not analyzed and thus the rate of upstaging is lower.

In addition to upstaging, we would like to briefly discuss the influence of the lymphvascular space invasion (L1). This was the only factor, which is associated with worser PFS. So far little is known about the significance of the L1 status in early stage NSCLC. It is still under continuous debate. There are only a few studies that have described L1 as a significantly associated factor with early tumor recurrence and independently associated with the presence of regional LN involvement [29, 30]. Our analysis underscores the limited published data and may contribute to the debate on adjuvant treatment after lymphovascular space invasion.

Some limitations of this study have to be considered. First, our study is retrospective. Second, there could be bias by the surgens. To minimize bias like surgical preferences or the impact of patient’s health status and comorbidity we used the following approaches. By only including patients without LN involvement or metastases we excluded patients with a suspected higher tumor stage and patients with a higher operational risk and a higher likelihood of thoracotomy, which might have affected the outcomes in our study. The primary goal of using PSM was to mitigate potential selection bias by balancing baseline characteristics between the two surgical groups, as patients undergoing VATS may differ systematically from those undergoing thoracotomy in ways that could confound the results. Additionally, we used multivariate regression analyses to adjust for known confounders. Nevertheless, we cannot exclude bias from surgeons deciding that patients with more comorbidities should have thoracotomy in order to achieve e.g. a shorter operating time. This would mean that the thoracotomy group contains sicker patients. Third, other confounding variables that may influence the results are the expertise of the surgeons and the variability of lymphadenectomy techniques. However, to address surgeons’ expertise to some extent, we added an analysis for a subgroup of patients with surgery from 2017 onwards. Regarding variability in lymphadenectomy, only patients in whom lymphadenectomy was performed according to the guidelines were included, so the variability is minimized.

## Conclusion

We were able to show that after standardized lymph node dissection, lymph node upstaging as a surrogate marker is independent of the surgical approach. If a standardized lymphadenectomy is performed, this results in a large number of removed LN and a representative upstaging. However, this technique requires the necessary expertise.

We were able to achieve LN upstaging of 12–18% in preoperatively expected N0 patients, which has a major impact on further therapy and PFS.

In future research, we recommend validating our results in multicenter randomized controlled trials. It would be important to conduct studies that perform a standardized lymphadenectomy in both cohorts (thoracotomy and VATS) in order to obtain representative results.

## Data Availability

Data from 1691 patients were recorded in the database. This data is still being analyzed and further studies are in progress. In order not to jeopardize the data for the new studies, they cannot be disclosed at this time. We ask for your understanding. The data sets and materials of the current study are available on reasonable request from the corresponding author.

## Abbreviations

ACC: Adenocarcinoma
ASA: American society of anesthesiologists risk classification
BMI: Body mass index
CCI: Charlson comorbidity index
FEV1: Forced expiratory volume in 1 s
HR: Hazard ratio
IASLC: International association for the study of lung cancer
LN: Lymph node
NEC: Neuroendocrine carcinoma
NSCLC: Non-small cell lung cancer
OS: Overall survival
OR: Odds ratio
PFS: Progression free survival
SCC: Squamous-cell carcinoma
sd: Standard deviation
VATS: Video-assisted thoracoscopic surgery
